# Micro-Chemistry of Poisons

**Published:** 1885-07

**Authors:** 


THE MICRO-CHEMISTRY OF POISONS. *By* THEODORE G. WORMLEY, M. D., PH. D., LL. D. *Philadelphia:* J. B. LIPPINCOTT. *Chicago:* W. T. KEENER, *96 Washington Street.*

A new edition of a work as well known as Professor Wormley's Micro-Chemistry of Poisons cannot fail to attract a good deal of attention from those interested in that special branch of chemical investigation. The second edition, which appeared a few months ago, is distinguished from the first, chiefly by the larger amount of matter it contains, and the addition of several new plates. These plates constitute, of course, the characteristic feature of the work, and their history is too well known to need an explanation here. But while being in a certain sense the characteristic, they are far from being the most important part of the book, and it is perhaps not far from the truth to say that without them the manual would possess nearly the same *practical* value, although the term "micro," as used by the author, could no longer be applied to it.

A book of no little interest could be written on the use and abuse of the microscope, and in the present instance, it seems to me, we have an illustration of how far one's enthusiasm may carry him in the choice of means not always best adapted to the end to be attained. The microscope renders us invaluable assistance in many investigations, often deciding the nature of a substance where chemical methods utterly fail; but any one who has had experience in the examination of microscopic crystals must understand the difficulties in the way of obtaining characteristic forms, and how far these difficulties are increased when the material to be tested has first to be separated from complex organic mixtures.

Where chemical and physiological tests fail, there is certainly little use in trying to discriminate with the microscope, and in this connection the words of Woodman and Tidy are worth quoting:—"We consider it, therefore, unnecessary to enter into any descriptive detail, as others have done, of processes for the selection of millionths of a grain by such methods as the collection of microscopic sublimates, which we admit are as exquisitely beautiful as they are, in our opinion, totally unfitted for practical investigation. If such poisons

* * * cannot be detected by their reactions in a test-glass, we are doubtful whether we should ever be justified in giving positive evidence of their presence on a criminal trial, by their microscopic forms and microscopic reactions."— *Forensic Medicine, p.* 84.

I am inclined to think Professor Wormley himself recognizes the truth of this, as the larger part of his book is devoted to the discussion, not of microscopic forms, but of the regular chemical tests, methods of separation and physiological actions of the various poisons. One can speak only in terms of the highest praise of this part of the book, as also of that relating to the selection and discrimination of blood stains. It is interesting to note that Professor Wormley takes a very conservative view of the value of microscopic measurements in determining the nature of the blood corpuscles—a view which doubtless finds favor with all investigators, save those belonging to the ranks of the professional experts. J. H. L.

